# Liver Sinusoidal Endothelial Cells Contribute to Hepatic Antigen-Presenting Cell Function and Th17 Expansion in Cirrhosis

**DOI:** 10.3390/cells9051227

**Published:** 2020-05-15

**Authors:** Esther Caparrós, Oriol Juanola, Isabel Gómez-Hurtado, Amaya Puig-Kroger, Paula Piñero, Pedro Zapater, Raquel Linares, Fabián Tarín, Sebastián Martínez-López, Jordi Gracia-Sancho, José M. González-Navajas, Rubén Francés

**Affiliations:** 1 Departamento Medicina Clínica, Universidad Miguel Hernández, 03550 San Juan de Alicante, Spain; ecaparros@umh.es (E.C.); ojuanola@gmail.com (O.J.); raquelinares.21@gmail.com (R.L.); sebastian.martinez@goumh.umh.es (S.M.-L.); 2 CIBERehd, Instituto de Salud Carlos III, 28029 Madrid, Spain; isanerea@hotmail.com (I.G.-H.); zapater_ped@gva.es (P.Z.); jordi.gracia@idibaps.org (J.G.-S.); gonzalez_josnav@gva.es (J.M.G.-N.); 3 Instituto de Investigación Sanitaria Gregorio Marañón (IiSGM), Hospital General Universitario Gregorio Marañón, 28007 Madrid, Spain; amaya.puig@iisgm.com; 4 Instituto ISABIAL, Hospital General Universitario Alicante, 03010 Alicante, Spain; paupinrom@gmail.com (P.P.); tarin_fab@gva.es (F.T.); 5 Departamento Farmacología, Universidad Miguel Hernández, 03550 San Juan de Alicante, Spain; 6 Instituto de Investigaciones Biomédicas August Pi i Sunyer (IDIBAPS), 08036 Barcelona, Spain

**Keywords:** cirrhosis, liver sinusoidal endothelial cells, dendritic cells, hepatic macrophages, Th cells, norfloxacin

## Abstract

Hepatic immune function is compromised during cirrhosis. This study investigated the immune features of liver sinusoidal endothelial cells (LSECs) in two experimental models of cirrhosis. Dendritic cells, hepatic macrophages, and LSECs were isolated from carbon tetrachloride and bile duct-ligated rats. Gene expression of innate receptors, bacterial internalization, co-stimulatory molecules induction, and CD4+ T cell activation and differentiation were evaluated. Induced bacterial peritonitis and norfloxacin protocols on cirrhotic rats were also carried out. LSECs demonstrated an active immunosurveillance profile, as shown by transcriptional modulation of different scavenger and cell-adhesion genes, and their contribution to bacterial internalization. LSECs significantly increased their expression of CD40 and CD80 and stimulated CD4+ T cell activation marker CD71 in both models. The pro-inflammatory Th17 subset was expanded in CCl_4_-derived LSECs co-cultures. In the bile duct ligation (BDL) model, CD4+ T cell differentiation only occurred under induced bacterial peritonitis conditions. Differentiated pro-inflammatory Th cells by LSECs in both experimental models were significantly reduced with norfloxacin treatment, whereas Foxp3 tolerogenic Th CD4+ cells were expanded. Conclusion: LSECs’ participation in the innate-adaptive immune progression, their ability to stimulate pro-inflammatory CD4+ T cells expansion during liver damage, and their target role in norfloxacin-induced immunomodulation granted a specific competence to this cell population in cirrhosis.

## 1. Introduction

The development and progression of cirrhosis are associated with an immunological dysfunction as one major factor in the increased susceptibility to bacterial infections, a frequent and clinically relevant complication in the evolution of cirrhotic patients [1]. This (endogenous) bacterial load is delivered into the liver through the portal circulation and, even in the absence of overt infection, the hepatic immune system continuously faces gut-derived antigens that require efficient clearance to avoid disease complications [2]. These events call attention to the cells responsible for hepatic immune surveillance in the context of liver failure.

Hepatic antigen presentation is professionally conducted by dendritic cells (DCs) and hepatic macrophages (HMs) [3,4]. In the steady-state, these antigen-presenting cells (APCs) are responsible for maintaining intrahepatic immune tolerance, restraining T cell activation, and promoting regulatory T cell differentiation [5]. During cirrhosis and probably further boosted due to the gut microbiota shift towards pathogenic clusters [6,7], hepatic APCs mediate the transition to a persistent immune activation and a pro-inflammatory T cell response [8,9,10,11]. Thus, the different receptors are responsible for the clearance of continuously incoming pathogenic antigens and complement intermediaries, and for leukocyte adhesion and infiltration to inflamed tissues when recruited [4].

Liver sinusoidal endothelial cells (LSECs) are involved in metabolic functions, such as lipids and lipoproteins transport [12,13], and in clearing waste from the blood due to their strong endocytic and lysosomal capacities [14]. Immunologically, studies have reported that LSECs serve as APCs in the tolerogenic liver [5,15]. During inflammation, LSECs recruit leukocytes through differential expression of adhesion molecules in specific liver diseases and cancer [16].

However, there is scarce data on LSEC immune functions in cirrhosis and, particularly, their ability to differentiate an effector T cell response in this context. The aim of the present study was to compare antigen-presenting and T cell differentiation features of LSECs with immune professional hepatic APCs in two different models of experimental cirrhosis. Besides, as frequent situations in cirrhosis, we also investigated the LSEC profile in induced bacterial peritonitis (iBP) and intestinal decontamination with norfloxacin.

## 2. Materials and Methods

### 2.1. Animals and Study Protocol

Male Sprague-Dawley rats (Harlan, Barcelona, Spain) weighing 200–250 g were caged at a constant room temperature of 21 °C, exposed to a 12:12 light/dark cycle, and fed with standard rodent chow, according to the study protocol.

Animals were subjected to carbon tetrachloride (CCl_4_, *n* = 45) and bile duct ligation (BDL, *n* = 45) protocols to induce experimental cirrhosis. Thirty-five CCl_4_ and 30 BDL animals completed the experimental protocols. Briefly, the CCl_4_ protocol was performed by administering weight-controlled doses of CCl_4_ intragastrically, as previously described for a period of 16 weeks [17]. A subgroup of animals acted as CCl_4_ controls and received mineral oil for 16 weeks (*n* = 12). BDL surgery was carried out by ligation of the common bile duct, as described elsewhere [18]. After surgeries, animals then started a 4-week protocol to develop experimental cirrhosis. A subgroup of animals acted as BDL controls and were sham-operated (*n* = 12).

Animals were sacrificed when severely ill, and death was suspected to be imminent. Twenty-four hours before laparotomies, a subgroup of naïve control rats (*n* = 12) and animals from both cirrhosis protocols (*n* = 10–12/protocol) received *Escherichia coli* (serotype 0111:B4) (10^7^ CFU/ip) to drive induced bacterial peritonitis (iBP). Twelve naïve rats remained untreated as controls. One week before laparotomies, the second subgroup of animals in both cirrhotic protocols (*n* = 10–12/protocol) received daily doses of norfloxacin (5 mg/kg/d) by gavage [19].

At laparotomy, blood (2 mL) from the vena cava was inoculated under aseptic conditions in sterile, rubber-sealed Vacutainer SST II tubes (BD Diagnostics, Temse, Belgium) that were never exposed to free air. All detectable mesenteric lymph nodes (MLNs) from the ileocecal area were removed under aseptic conditions and liquefied in sterile saline for bacterial culture. MLNs were homogenized by sonication, and one aliquot of the homogenate was cultured in chromogenic aerobic media (CrhomID-CPS3, Biomerieux, Marcy l’Etoile, France) and incubated at 37 °C. After 24–48 h, colonies were identified. Spleens from all rats were collected in RPMI 1640 (Thermo Fisher, Waltham, MA, USA), 10% fetal bovine serum supplemented with 1% penicillin/streptomycin and 1% L-glutamine (RP10) prior to liver perfusion in situ with Hanks’ balanced salt solution (HBSS) without Ca^2+^ and Mg^2+^ at 37 °C. This was followed by perfusion with HBSS containing 100 mM CaCl_2_ solution at the same perfusion rate. The liver was then removed and rinsed with HBSS. Liver biopsy specimens, 10–15 mm in size, were collected and conserved in RNA later (Sigma-Aldrich, San Luis, MO, USA). Animals were then euthanized by an overdose of anesthesia. A complete study protocol can be found in Figure S1.

Animals’ handling and care were performed according to the criteria outlined in the Guide for the Care and Use of Laboratory Animals. The study was approved by the Animal Research Committee of Universidad Miguel Hernández (2016/VSC/PEA/00081) (Alicante, Spain).

### 2.2. Liver APCs Isolation

Hepatic DCs, HMs, and LSECs were isolated from all animals. Perfused livers were digested in vivo with collagenase A (Merck-Millipore, Burlington, MA, USA) in HBSS containing 12 mM HEPES and 4 mM CaCl_2_, as previously described [20]. Resultant digested livers were excised, and an in vitro digestion with the same buffer containing collagenase A was performed at 37 °C for 10 min. The liver cell solution was then filtered by using 100 µM nylon strainers and collected in cold Kreb’s solution containing 25 mM HEPES. The cell suspension was centrifuged at 50× *g* for 5 min, and parenchymal cells were separated by collecting the supernatants and then centrifuged at 800× *g* for 10 min. Resultant pellets were resuspended in 10 mL PBS.

APCs were enriched with a density gradient centrifugation at 800× *g* for 25 min by using Percoll 25% and 50%. Cells from the interphase were collected, washed with phosphate-buffered saline (PBS), and resuspended in PBS without Ca^2+^ and Mg^2+^ supplemented with 0.5% bovine serum albumin (BSA) and 2 mM ethylenediaminetetraacetic acid (EDTA) for magnetic separation. Cells were directly labeled with αOX-62 for DC selection and indirectly labeled with αCD68-PE and anti-PE microbeads for HM selection (Miltenyi Biotech, Madrid, Spain). For LSEC isolation, the negative elicited fraction was seeded in collagen-coated 6-well plates at a concentration of 10^6^ cells/mL per well and incubated for 45 min at 37 °C, 5% CO_2_ with RP10 supplemented with 1% fungizone, 1% endothelial cell growth supplement (ECGS), and 1% heparin. Cultured cells were washed twice with PBS and harvested with cell scrapers (VWR, Radnor, PA, USA). Cell viability evaluated by trypan blue was above 98% in all cases. Cells purity > 95% was confirmed by flow cytometry-specific labeling with αMHC-II for DCs (Biorad, Hercules, CA, USA), αCD163 for HMs (Origene, Rockville, MD, USA), and αCD32b for LSECs (Bioss, Woburn, MA, USA) (Figure S2).

### 2.3. Gene Expression Measurements

The transcriptional profile of a panel of innate immune receptors was analyzed in liver APCs. The main role of each evaluated receptor can be found in Table S1. RNA from isolated APCs (DCs, HMs, and LSECs) and liver tissue specimens was obtained with the RNeasy mini kit (Qiagen, Hilden, Germany) and quantified by using the spectrophotometer NanoDrop 1000 (Thermo Fisher Scientific, Waltham, MA, USA) for preparing 5 ng/µL dilutions.

We used 10 ng of total RNA from liver tissue for evaluating gene expression levels of collagen type 1 alpha-1 chain (COL1A1), transforming growth factor 1 (TGF1), and tissue inhibitor of metalloproteinase 1 (TIMP1). RNA from hepatic APCs was used for evaluating gene expression levels of complement component 3 (C3), complement component receptors (C5aR1, CR1, CR4), mannose receptor (CD206), co-stimulatory molecules (CD40, CD80, CD86), folate receptor beta (FOLR2), C-type lectin domain family 4-member G (CLEC4G or LSECtin), macrophage scavenger receptor 1 (MSR1), scavenger receptor class B member 1 (SCARB1), and toll-like receptors (TLR2 and TLR4). Samples were analyzed with qScript one-step SYBR green quantitative real-time PCR kit (Quanta BioScience, Gaithesburg, MD, USA) in an IQ5 real-time PCR (Bio-Rad, Hercules, CA, USA). All values were normalized with the housekeeping *gene β2-microglobulin* for gene expression analysis. For each gene and each cell population, mean 2^−ΔΔCt^ values were compared between animals in different study groups and represented as the percentage of variation between groups in paired comparisons. Primer-pairs used for mRNA analysis are described in Table S2. *P*-values from statistically significant comparisons are detailed in Figure S3.

### 2.4. In Vitro Phagocytosis and Killing Assays and Liver APCs’ Co-Stimulatory Molecules Expression

A total of 2.5 × 10^6^ liver-isolated APCs from different groups were incubated with the same amount of *E. coli* colony-forming units (CFUs) in the presence of autologous rat serum with HBSS in a final volume of 1 mL in an orbital shaker at 37 °C at 50 rpm. APCs and *E. coli* were incubated together for 20 min to measure baseline *E. coli*-binding and internalizing capacity of APCs (T_0_) and 2 h (T_2_) to measure APCs’ ability to kill *E. coli*. Cells were washed after incubations and subjected to a 30% sucrose centrifugation to separate non-bound *E. coli* from APCs. Resting APCs were resuspended in 1 mL HBSS 5% serum and diluted 1:5 in sterile water to release APC content. Dilutions were seeded in agar plates (Biomerieux, Marcy l’Etoile, France) and incubated overnight at 37 °C. CFUs were counted, and values were represented for each APC in the different study groups.

Cell surface expression of CD40-APC, CD80-PE, and CD86-FITC and their respective isotype controls (BD Biosciences, San Diego, CA, USA) was measured by flow cytometry in isolated DCs, HMs, and LSECs from animals in the different study conditions using a FACSCanto II flow cytometer operated by FACSDiva software (BD Biosciences, San Diego, CA, USA). Representative images of CD40, CD80, and CD86 and their isotype-matched controls are shown in Figure S4 for control rats.

### 2.5. Isolation of Splenic T Cells and Th Selection

Th CD4+ cells were isolated by immunomagnetic negative selection following the manufacturer’s instructions (EasySep Rat CD4+ Cell Isolation Kit, STEMCELL Technologies, Vancouver, Canada). Briefly, the spleen was disrupted in RP10 medium and filtered through a 70 µm-nylon strainer. The cell suspension was centrifuged, resuspended in HBSS without Ca^2+^ and Mg^2+^ containing 2% fetal bovine serum and 1 mM EDTA, and labeled with the isolation cocktail (50 µL/mL) for 10 min at room temperature. Afterward, RapidSpheres were added (50 µL/mL), mixed, and cell solution was placed in the magnet for specific negative isolation of purified Th CD4+ cells.

### 2.6. Co-Culture of APCs with Th CD4+ Cells and Flow Cytometry Staining

For the measurement of T cell activation, APCs and syngeneic Th CD4+ cells were plated in triplicate in 96-well round-bottom plates at 1:10 and incubated for 7 days in 5% CO_2_ atmosphere at 37 °C and subjected to extra or intracellular labeling. An ovalbumin (OVA) peptide-directed APC presentation to OVA receptor-specific expressing T cells approach was not considered as we intended to evaluate ex vivo T cell activation mediated by cirrhotic APCs already exposed to hepatic antigenic load along with their liver damage protocol. The αCD3-BV510 and αCD4-FITC antibodies were used to gate the specific Th CD4+ population. To measure Th CD4+ activation, cells were stained with αCD25-PECy7 and αCD71-PE antibodies (BD Biosciences). To evaluate the Th CD4 differentiation profile, cells were previously stimulated with phorbol myristate acetate/ionomycin (50 ng/mL and 1 µg/mL, respectively) (Sigma-Aldrich, Madrid, Spain) for 5 h, and Golgi traffic-blocked with monensin (BD Golgi STOP, BD Biosciences). Cells were then fixed and permeabilized, and intracellularly labeled with αFoxp3-PE for Treg population, αIFNg-BV421 for Th1, αIL-4 PE for Th2, and αIL-17-PerCPCy5.5 for Th17 population (Invitrogen, Carlsbad, CA, USA). Representative dot plots for the gating strategy of different CD4+ cells are depicted in Figure S5.

### 2.7. Statistical Analysis

Discrete variables were expressed as counts (percentage) and continuous variables following a normal distribution as mean ± standard deviation. To verify the normality of continuous variables, the Kolmogorov–Smirnov test (*p* < 0.05) was used. Differences between groups were analyzed using the Mann–Whitney U test for continuous variables. Multiple comparisons were analyzed according to Bonferroni correction. All reported *p*-values were two-sided, and *p*-values lower than 0.05 were considered to indicate significance (*p* < 0.008 for multiple comparisons). All calculations were performed using SPSS 19.0 software (IBM, Chicago, IL, USA).

## 3. Results

### 3.1. Animals

The CCl_4_ and BDL protocols showed mortality rates of 22% (10/45 rats) and 33% (15/45 rats), respectively. Ascitic fluid was present in 40% (14/35) of CCl_4_ rats and 26% (8/30) of BDL rats. Gene expression levels of profibrogenic markers were evaluated in all rats from both protocols (Figure S6). Positive microbiological cultures were present in MLNs from two out of six rats in the CCl_4_ group without norfloxacin or *E. coli* (*Streptococcus viridians S. viridans*, *E. coli*) and from two out of six rats in BDL rats without norfloxacin or *E. coli* (*Klebsiella pneumoniae, Shigella flexneri K. pneumoniae*, *S. flexneri*). All animals in groups administered with *E. coli* ip. showed positive microbiological cultures in MLNs, whereas none of the cirrhotic animals treated with norfloxacin did.

### 3.2. LSECs Displayed Immunosurveillance Markers during Experimental Liver Damage

**Figure 1:**
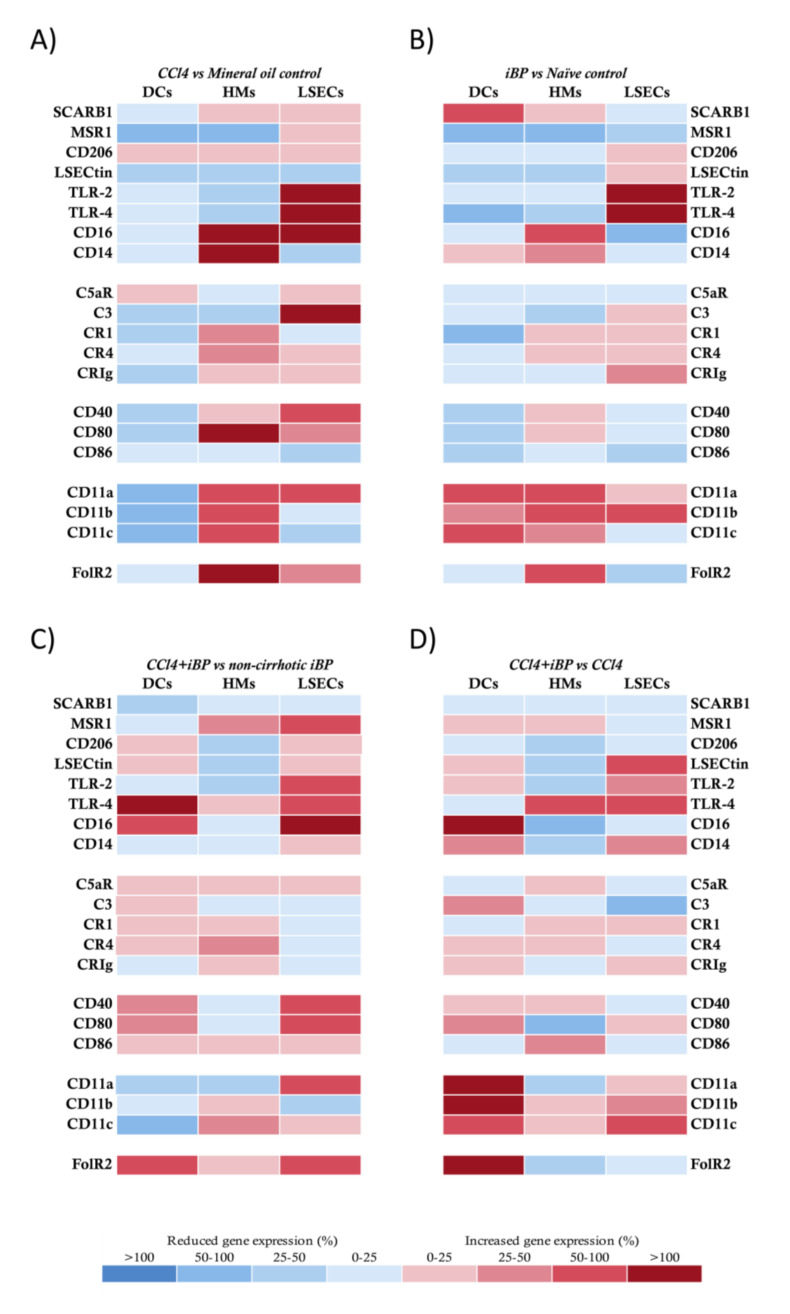
Transcriptional profile comparison of innate receptors in antigen-presenting cells (APCs) from CCl_4_ animals. (**A**) CCl_4_-treated vs. mineral oil control animals, (**B**) iBP treated animals vs. naïve control group, (**C**) CCl_4_+iBP treated vs. non-cirrhotic iBP group, and (**D**) CCl_4_+iBP animals vs. CCl_4_ group. Differential expression of up (red) and downregulated (blue) genes is scaled according to the color depicted. For each gene and each cell population, mean 2^−ΔΔCt^ values were compared between animals in different study groups and represented as the percentage of variation between groups in paired comparisons. SCARB1: scavenger receptor class B member 1; MSR1: macrophage scavenger receptor 1; LSECtin: liver and lymph node sinusoidal endothelial cell C-type lectin; TLR: toll-like receptor; CR: complement receptor; CRIg: complement receptor of the immunoglobulin superfamily; FolR2: folate receptor 2; DC: dendritic cell; HM: hepatic macrophage; iBP: induced bacterial peritonitis; LSEC: liver sinusoidal endothelial cell.

The expression of innate receptors is a hallmark of APC functionality. We first evaluated this expression in LSECs in comparison to HMs and DCs. In the CCl_4_ model, DCs showed a global protolerogenic state compared to control rats, while HMs and LSECs displayed a general increment in the transcriptional receptor profile. Particularly, both HMs and LSECs increased their expression levels of co-stimulatory molecules CD40 and CD80 and different complement and integrin receptors. In addition, HMs from CCl_4_ rats significantly increased the expression of CD14, CD16, and FolR2, and LSECs increased the expression of CD16, TLR2, and TLR4 compared to control animals (Figure 1A).

Next, we set out to evaluate whether the response to iBP in cirrhosis might be similar to that in control rats. To do so, we first compared the effect of iBP on non-cirrhotic control rats. In response to a bacterial insult, DCs in control rats increased the expression of the different components of the integrin receptors and SCARB1, although most markers were not transcriptionally upregulated. HMs and LSECs in control rats upregulated a number of innate receptors in response to iBP (Figure 1B). The response of CCl_4_ cirrhotic animals to iBP versus non-cirrhotic iBP rats revealed that DCs showed a mild general increase in gene expression levels of different receptors and a particular significant upregulation of TLR4, CD16, and FolR2 receptors. While HMs did not show a significant upregulation of studied receptors, LSECs from CCl_4_ cirrhotic animals sustained higher levels of MSR1, TLRs, CD16, and co-stimulatory molecules compared to control rats under iBP conditions (Figure 1C).

We then compared whether iBP was able to induce an additional effect to that caused by the CCl_4_ background on the transcriptional activity of innate receptors. DCs shifted their transcriptional profile, and, according to the acute bacterial insult, gene expression levels of the recruiting and cell adhesion integrin components—CD11a, b, and c—were significantly increased. HMs showed a mild modulation of several receptors in response to the induced bacterial challenge, whereas LSECs further increased the transcriptional profile of LSECtin, TLRs, and CD11c in this context (Figure 1D).

**Figure 2:**
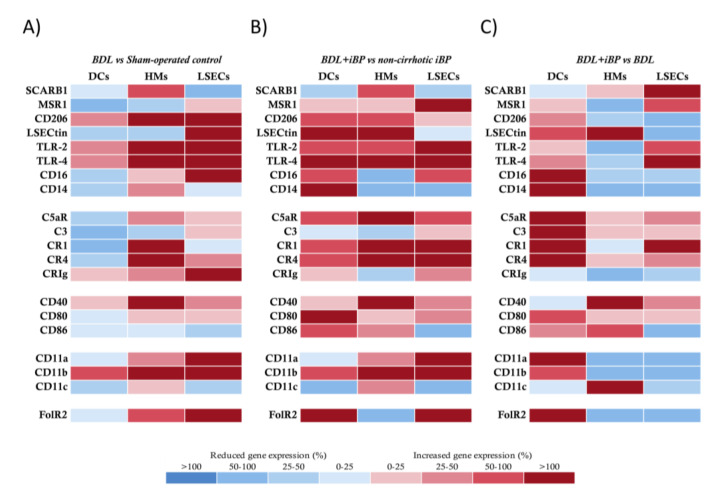
Transcriptional profile comparison of innate receptors in APCs from bile duct ligation (BDL) animals. (**A**) BDL group vs. sham-operated control animals, (**B**) BDL+iBP animals vs. non-cirrhotic iBP group, and (**C**) BDL+iBP group vs. BDL group. Differential expression of up (red) and downregulated (blue) genes is scaled according to the color depicted. For each gene and each cell population, mean 2^−ΔΔCt^ values were compared between animals in different study groups and represented as the percentage of variation between groups in paired comparisons.

The BDL model strongly induced the upregulation of most of the innate receptors’ gene expression in HMs and LSECs, whereas DCs remained in a protolerogenic state, as previously observed in the CCl_4_ model (Figure 2A). In agreement, DCs from BDL rats with iBP increased the transcriptional activity of analyzed receptors either compared to control rats with iBP (Figure 2B) or to BDL rats (Figure 2C). HMs and LSECs in the BDL model upregulated the expression of antigen recognition and binding markers, co-stimulatory molecules, and integrin components in response to iBP compared to control rats with iBP (Figure 2B). When the effect of iBP was evaluated in BDL rats, we observed that HMs only upregulated LSECtin and co-stimulatory molecules, while the gene expression levels of antigen recognition markers and integrin components—CD11a and CD11b—were significantly decreased. LSECs retained the ability to induce complement receptor, TLR, and scavenger receptors’ transcriptional activation, while, similarly to HMs, the integrin components—CD11a, b, and c—were significantly downregulated in response to iBP among BDL rats (Figure 2C).

### 3.3. LSECs Significantly Cooperated in E. coli Internalization and Killing Activity in Experimental Cirrhosis

**Figure 3:**
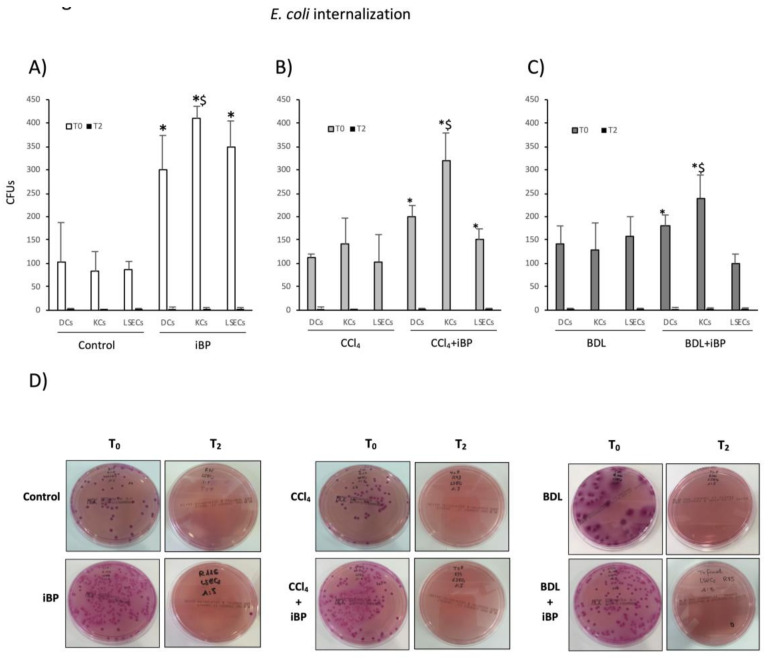
*E. coli*-binding and internalizing capacity of APCs. (**A**–**C**) *E. coli*-binding and internalizing capacity of APCs was measured by counting colony-forming units (CFUs) at baseline (T_0_). To measure APCs’ ability to kill uptaken bacteria, CFUs were counted after 2 h incubation at 37 °C (T_2_). Mean values were represented for each APC in control vs. iBP rats (**A**), CCl_4_ vs. CCl_4_+iBP (**B**), and BDL vs. BDL+iBP (**C**). (**D**) Representative plates of *E. coli* CFUs grown at T_0_ and T_2_ in pre-incubated LSECs with *E. coli*. * *p* < 0.01 compared with the same cell type at T_0_ in groups without iBP; ^$^ *p* < 0.01 compared with other cell types at T_0_ in the same group.

Phagocytic activity is also an important signature of APCs. To determine whether LSECs were able to perform such activity, hepatic APCs’ ability to uptake (T_0_) and kill (T_2_) *E. coli* in vitro was evaluated in experimental cirrhosis. A previous bacterial challenge (iBP condition) in control rats stimulated DCs, HMs, and LSECs’ capacity to uptake *E. coli* (Figure 3A). DCs and HMs from cirrhotic animals, either CCl_4_ or BDL, were able to uptake *E. coli* in vitro as efficiently as control animals. LSECs exhibited similar behavior to that shown by DCs and HMs, both from CCl_4_-treated (Figure 3B) and BDL animals (Figure 3C), being a significant contributing cell type in the hepatic antigen clearance function during experimental liver injury. LSECs also contributed to the marked HM internalizing capacity in response to acute bacterial challenge in the cirrhotic background (Figure 3B,C). Figure 3D shows representative plate images of LSEC bacterial internalization (T_0_) and killing (T_2_) capacities in the different experimental conditions.

### 3.4. LSECs Participated in Bridging Innate and Adaptive Immunity during Experimental Cirrhosis

Co-stimulatory molecules signaling is indispensable for the correct initiation of the T cell-specific immune response. The aim was to determine the capacity of LSECs to express these co-stimulatory molecules, signaling the expression of CD40, CD80, and CD86 in liver APCs from the different liver damage models.

**Figure 4:**
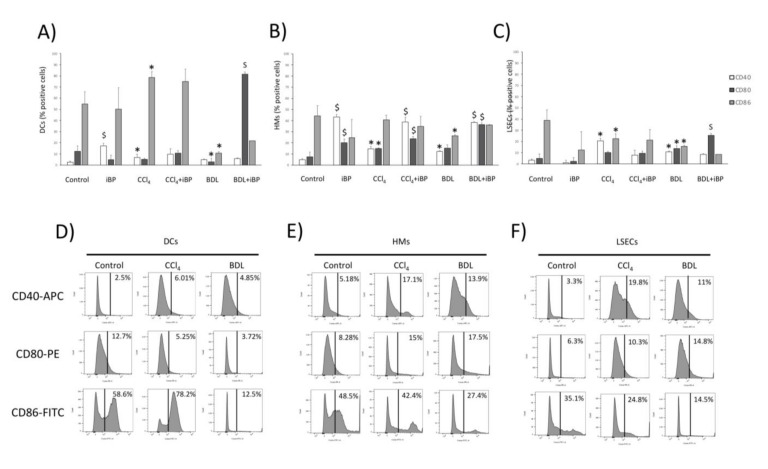
Cell membrane expression of co-stimulatory molecules in hepatic APCs. Cell membrane expression of co-stimulatory molecules CD40, CD80, and CD86 evaluated by flow cytometry in DCs (**A**), HMs (**B**), and LSECs (**C**) from control (*n* = 6), CCl_4_ (*n* = 6), and BDL (*n* = 6) animals in different experimental conditions. (**D**–**F**) Representative histograms, showing the expression of co-stimulatory molecules in DCs (**D**), HMs (**E**), and LSECs (**F**) in control rats and the two experimental models of cirrhosis. * *p* < 0.01 compared to control rats; ^$^ *p* < 0.01 compared with the same group without iBP.

In DCs, the percentage of CD40-expressing cells significantly increased in iBP compared to control, while in CCl_4_ and BDL groups, no significant variations were observed in this marker among different liver damage conditions. The percentage of DCs expressing CD80 was only upregulated in BDL rats under the iBP challenge. The CD86 marker was expressed in a significantly higher number of DCs of animals treated with CCl_4_, whereas in the case of BDL, this molecule was significantly lower than in control animals (Figure 4A). In HMs, the expression of CD40 and CD80 significantly increased in response to iBP, irrespective of the presence of liver damage, while CD86 remained unaltered (Figure 4B). The percentage of LSECs expressing CD40 was higher in CCl_4_ rats compared to control and BDL rats. CD80 was significantly increased in CCl_4_ and BDL rats compared to controls, and CD86 was not further upregulated in LSECs from animals under liver damage (Figure 4C). Cell membrane expression of co-stimulatory molecules in mineral oil-administered and sham-operated controls showed no significant differences compared to naïve control rats (data not shown). Representative histograms for CD40, CD80, and CD86 in DCs (Figure 4D), HMs (Figure 4E), and LSECs (Figure 4F) are depicted for control, CCl_4_, and BDL rats.

### 3.5. LSECs Stimulated the Activation and Differentiation of the Adaptive T CD4+ Cell Response in Rats with Cirrhosis

**Figure 5:**
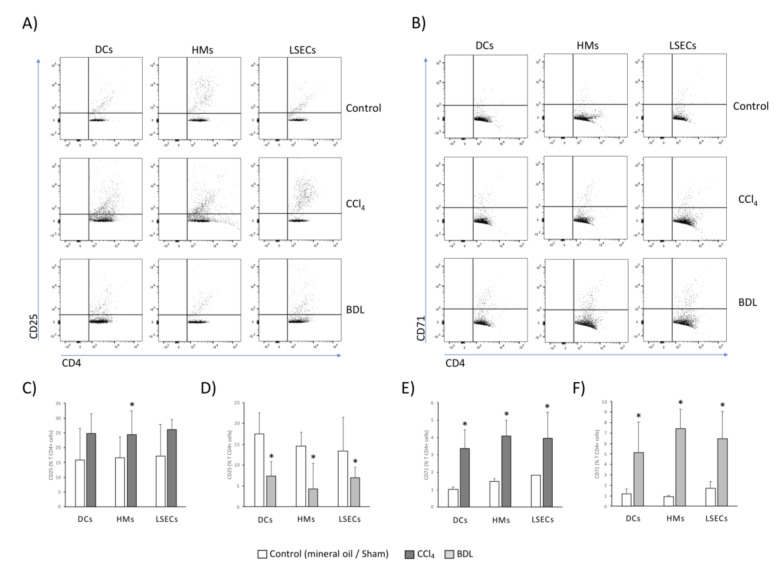
Activation marker expression in spleen-derived T CD4+ cells after co-culture with hepatic APCs. Dot plot images for CD25 (**A**) and CD71 (**B**) in CCl_4_ and BDL models. Mean values ± standard deviation of percentage of T CD4+ cells expressing CD25 in CCl_4_ (**C**) and BDL models (**D**), and CD71 in CCl_4_ (**E**) and BDL models (**F**), are represented from at least 5 animals per group. * *p* < 0.01 compared with the same cell type in the control group.

A relevant function of APCs is the promotion of an appropriate T cell response depending on the tissue microenvironment context. To determine LSECs capacity to undertake this process, APCs were co-cultured with Th cells, and their specific capacity to activate the adaptive T CD4+ response was evaluated. DCs and HMs isolated from the liver of CCl_4_-induced cirrhotic animals induced the expression of CD25 (Figure 5A) and CD71 (Figure 5B), markers of T CD4+ cell activation. Likewise, LSECs induced significant activation of Th cells, comparable to that of DCs and HMs. In the BDL model, DCs, HMs, and LSECs induced the expression of CD25 only a fourth to a third as much as in CCl_4_-treated animals (Figure 5C,D), whereas all APCs were able to stimulate a similar percentage of T CD4+ CD71 expression to that in the CCl_4_ background (Figure 5E,F). To confirm hepatic APCs capacity to activate Th cells, control conditions with lipopolysaccharide (LPS) pre-treated cells were included in both cases (data not shown).

**Figure 6:**
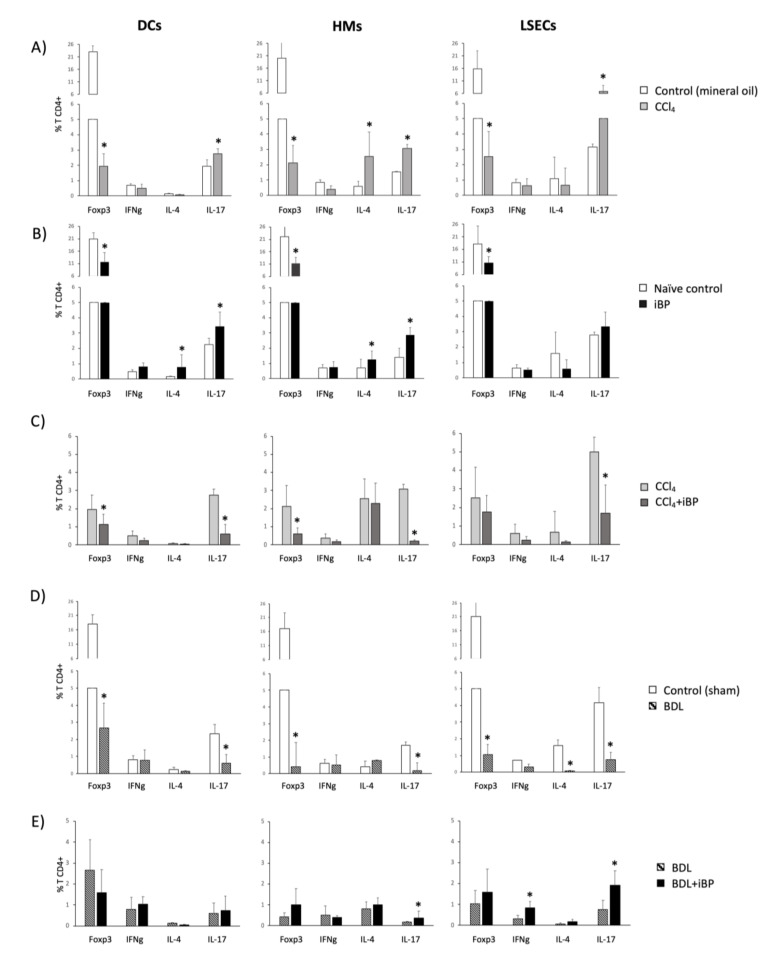
Intracellular expression of Th differentiation markers in spleen-derived T CD4+ cells after co-culture with hepatic APCs. Mean values ± standard deviation of percentage of T CD4+ cells expressing Forkhead box p3 (Foxp3) (Treg), IFNg (Th1), IL-4 (Th2), and IL-17 (Th17) are represented comparing CCl_4_ vs. mineral oil-treated control (**A**), iBP vs. control (**B**), CCl_4_ vs. CCl_4_+iBP (**C**), BDL vs. sham-operated control (**D**), and BDL vs. BDL+iBP (**E**) groups. All groups included at least 5 animals. * *p* < 0.01 compared with each marker in the other represented group.

The T CD4+ cell tolerogenic profile shown by control rats, evidenced by the significant Foxp3 expression over the Th-specific cytokines, shifted towards a proinflammatory polarized environment as a consequence of the induced toxic CCl_4_ liver damage, biased to a prominent Th17 differentiation in the case of DCs and LSECs, and to a Th2/Th17 co-differentiation profile in the case of HMs, with a drastic downregulation of CD4+ Treg population. Notably, LSECs were able to double the percentage of differentiated Th17 cells compared to the other APCs studied (Figure 6A).

An acute iBP challenge in control rats stimulated DCs and HMs to induce an important Th2 and Th17 programming, while LSECs only induced a mild increase in the Th17 differentiation profile (Figure 6B). Interestingly, the iBP challenge in the CCl_4_-treated animals revealed that neither DCs nor HMs or LSECs could further promote an additional T CD4+ cell response to an acute insult. Conversely, the T CD4+ cell differentiation was even lessened (Figure 6C).

In the BDL model, the Th17 population was significantly downregulated in the co-cultures with DCs, HMs, or LSECs. APCs showed the capability to promote neither the Th1 nor the Th2 phenotype in the co-cultured T cells compared to controls (Figure 6D). According to this result, and in contrast to what was observed in CCl_4_, the iBP challenge in BDL rats significantly initiated the differentiation of Th17 cells by HMs and of Th1 and Th17 cells by LSECs (Figure 6E).

As supplementary data to support the induction of Th17 by LSECs, we evaluated IL-6 and TGF-β mRNA expression levels in isolated cells from all studied groups of animals and also measured IL-17 in the supernatants of co-cultured Golgi-traffic unblocked LSEC/CD4+ T cells. Results confirming increased IL-6 and TGF-β mRNA levels in LSECs and increased IL-17 levels in the supernatants of unblocked co-cultures supported LSECs participation in Th17 response (Figure S7).

### 3.6. Norfloxacin Limited the Activation of Innate Immune Receptors and Preserved LSECs Functionality, Favoring LSEC-Dependent Treg Differentiation

**Figure 7:**
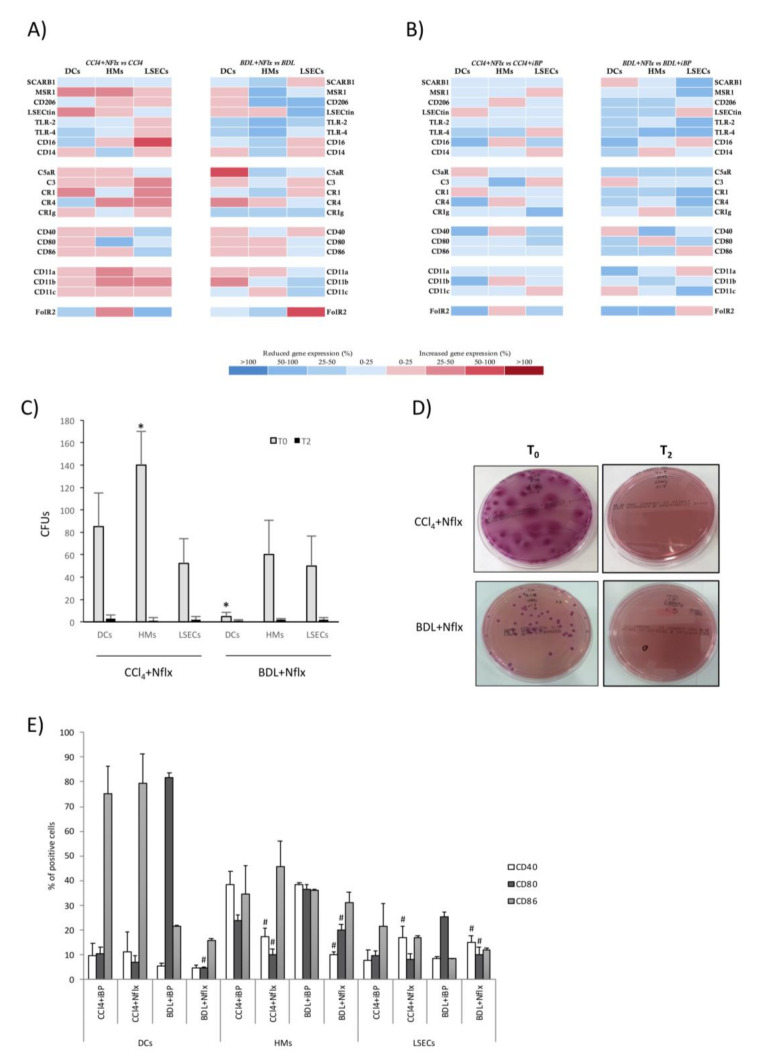
APC analyses in animals treated with norfloxacin. Transcriptional profile representation of innate receptors in (**A**) antigen-presenting cells from CCl_4_ and BDL animals with and without norfloxacin, and (**B**) norfloxacin-treated CCl_4_ and BDL animals with and without iBP. Differential expression of up (red) and downregulated (blue) genes is scaled according to the color depicted. (**C**) Internalization of *E. coli* by hepatic APCs from CCl_4_ and BDL animals treated with norfloxacin. *E. coli*-binding and internalizing capacity of APCs was measured by counting CFUs at baseline (T_0_). To measure APCs’ ability to kill uptaken bacteria, CFUs were counted after 2 h incubation at 37 °C (T_2_). (**D**) Representative images of CFUs grown after internalization assay in LSECs. (**E**) The expression of co-stimulatory molecules—CD40, CD80, and CD86—evaluated by flow cytometry in APCs from CCl_4_ and BDL animals either with iBP or norfloxacin treatment. All experiments performed in 5 animals per group. * *p* < 0.01 compared with other cell types at T_0_ in the same group. *^#^ p* < 0.01 compared with the corresponding iBP group.

Norfloxacin is a fluoroquinolone commonly used for selective intestinal decontamination as secondary prophylaxis of spontaneous bacterial peritonitis. We investigated all previously evaluated LSECs properties in cirrhotic rats with norfloxacin and compared it to acute damage and acute-on-chronic liver damage models. The use of norfloxacin in CCl_4_ and BDL rats was not associated with a significant increase in any of the evaluated receptors for any of the three populations compared with cirrhotic rats without norfloxacin (Figure 7A). The comparison between norfloxacin-treated versus iBP-administered CCl_4_ and BDL animals revealed an overall mild reduction of the transcriptional profile of most of the innate receptors studied in all three populations (Figure 7B).

Internalizing capacity remained active in hepatic APCs from norfloxacin-administered animals (Figure 7C). Figure 7D shows representative plate images of LSEC bacterial internalization (T_0_) and killing (T_2_) capacities in the different experimental conditions. Norfloxacin-administered versus non-administered CCl_4_ and BDL animals did not change their membrane expression of co-stimulatory molecules in DCs, HMs, or LSECs (see control, iBP, CCl_4_, and BDL levels in Figure 4). However, membrane expression of CD40 was downregulated in HMs from CCl_4_ and BDL animals with norfloxacin compared with iBP-administered cirrhotic animals, while this marker in LSECs was upregulated. The percentage of CD80+ cells in HMs from CCl_4_ animals and in all APCs from BDL animals with norfloxacin was significantly lower than iBP-administered CCl_4_ and BDL animals, respectively. No significant differences between groups were observed in the number of APCs expressing co-stimulatory molecule CD86 (Figure 7E).

**Figure 8:**
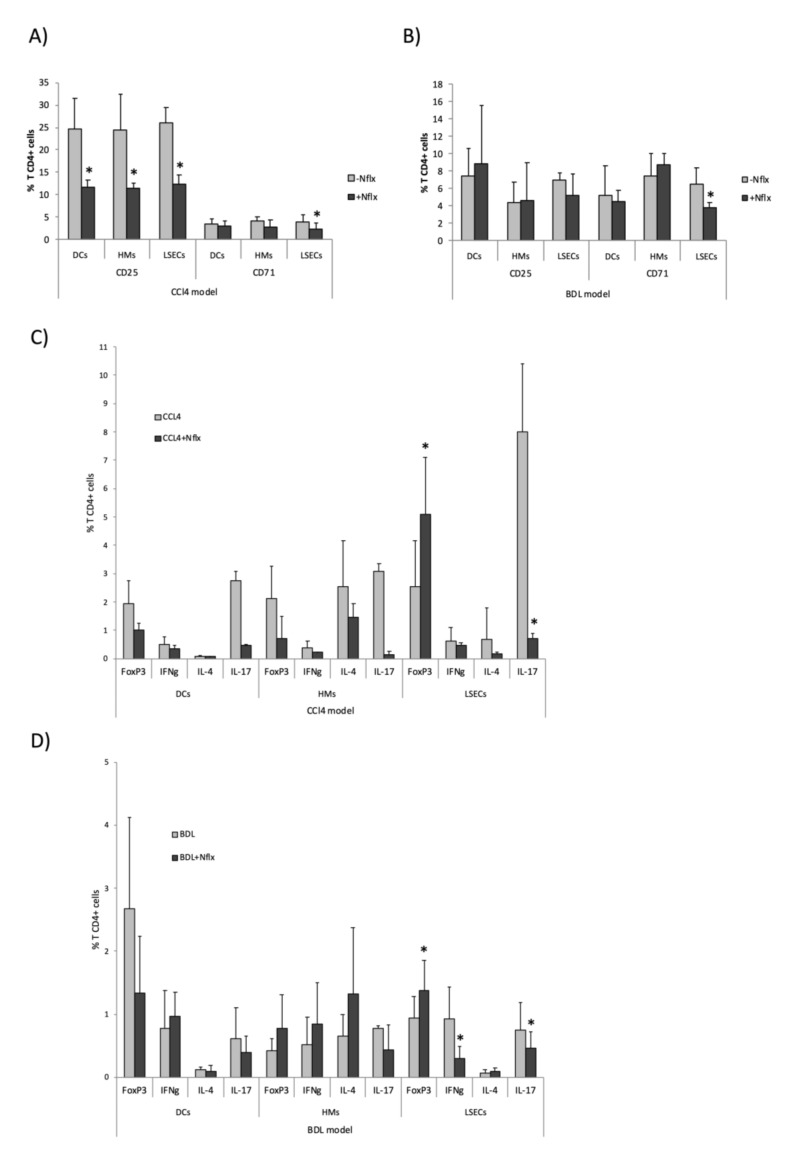
Activation and differentiation markers in spleen-derived T CD4+ cells co-cultured with APCs. The expression of activation and differentiation markers in spleen-derived T CD4+ cells after co-culture with hepatic APCs in CCl_4_ and BDL animals administered or not with norfloxacin. Percentage of T CD4+ cells expressing CD25 and CD71 in CCl_4_ model (**A**) and BDL model (**B**). Percentage of T CD4+ cells expressing Foxp3 (Treg), IFNg (Th1), IL-4 (Th2), and IL-17 (Th17) in CCl_4_ model (**C**) and BDL model (**D**). Mean values ± standard deviations are represented from at least 5 animals per group. * *p* < 0.01 compared with norfloxacin-untreated animals.

Similarly, hepatic APCs from CCl_4_ animals treated with norfloxacin were able to activate T CD4+ cells, as observed by the percentage of CD25- and CD71-expressing Th cells. However, this capacity was lower than that in APCs from CCl_4_ animals without norfloxacin (Figure 8A). In the BDL model, hepatic APCs’ capacity to activate Th cells was not significantly modified by norfloxacin administration, except for CD71 by LSECs (Figure 8B). The percentage of differentiated Th cells by APCs in the experimental models of liver disease was significantly reduced with the norfloxacin treatment in the CCl_4_ model (Figure 8C), whereas it didn’t induce significant changes in the BDL model, except for IFNg and IL-17 by LSECs (Figure 8D). In addition, LSECs were able to induce Foxp3 tolerogenic Th CD4+ cells in CCl_4_ (Figure 8C) and BDL (Figure 8D) models.

All experimental procedures were also conducted in a subgroup of female rats. No significant differences were present for any of the variables evaluated (Figure S8).

## 4. Discussion

In the present study, we showed that LSECs were versatile antigen-presenting cells able to differentiate a CD4+ Th17 adaptive response in the inflammatory context of CCl_4_-induced experimental cirrhosis. LSECs modulated their innate receptor’s transcriptional profile, bridged innate and adaptive immunity by inducing the expression of co-stimulatory molecules, cooperated in phagocytic tasks, and activated CD4+ T cells to differentiate effector Th responses in cirrhosis. LSECs also kept their immune collaborative function by responding under acute bacterial challenge conditions and in selective decontamination with norfloxacin.

LSECs are morphologically unique cells lining the liver, exposed to gut and blood-derived antigens [15]. Together with HMs, they constitute an efficient scavenger system [13,21]. The antigenic load arriving at the gut through the portal vein is increased in cirrhosis [2,22], highlighting the importance of innate hepatic receptor modulation to counteract this situation [23]. In agreement, we observed an increased transcriptional profile of scavenger receptors, co-stimulatory molecules, immune complexes receptors, and adhesion molecules in HMs and LSECs of CCl_4_ and BDL rats compared to control, even stronger than the profile induced against an acute bacterial insult in non-cirrhotic conditions. However, DCs remained in a protolerogenic state during induced liver damage, probably due to their described immature development status in the liver [24,25], which needed a second acute pro-inflammatory hit (as iBP in CCl_4_ and BDL backgrounds) in order to transcriptionally upregulate their innate receptors (Figure 1D and Figure 2C).

Although some studies have described LSECs’ APC properties, such as MHC-I and MHC–II or co-stimulatory molecules’ expression in basal conditions [26,27], we provided evidence that LSECs retained the capability of acting as APCs during cirrhosis as well. The phagocytic capacity of LSECs in the cirrhotic environment was similar to that observed for DCs and HMs, and at least as efficient as that observed in APCs from control livers. Furthermore, LSECs maintained their bacterial internalizing contribution in rats from both cirrhotic protocols with iBP (Figure 3). This result was supported by the known ability for LSEC to efficiently clear blood-borne LPS [28]. Nonetheless, LSECs showed a limited yet effective ability to uptake bacteria in cirrhotic vs. non-cirrhotic iBP animals. This reduction might be related to the pre-primed status caused by exposure to chronic inflammatory damage [1]. Despite LSECs have been described to ex vivo dedifferentiate a number of transcription factors and cytoskeletal-associated molecules while retaining others, such as the pan-endothelial marker CD31 [29], our data would sustain that antigen-presenting properties of LSECs are not altered in culture, either. LSECs’ APC features were supported by the surface expression of co-stimulatory molecules—CD40, CD80, and CD86 (Figure 4)—which might compromise their steady-state tolerogenic fate [30,31], qualifying this cell population for adaptive T cell activation under chronic inflammatory conditions.

An interesting result of the present study was LSECs’ ability to differentiate CD4+ T cells towards pro-inflammatory pathways in CCl_4_-induced cirrhosis (Figure 6). LSECs have been described to prime tolerance through CD8+ T presentation in low antigenic concentration [32,33] and even to activate CD4+ T cells to become regulatory and, therefore, protective while suppressing inflammatory CD4+ T cells in a model of T cell-mediated hepatitis [34,35]. However, the increased antigenic burden has been described to induce the shifting from tolerogenic to effector T cell differentiation in CD8+ T cells [36]. Although some exploratory results on LSEC-induced activation of CD4+ T cells in murine hepatic fibrosis have been previously described in a T CD8+ cell-specific activation centered study [37], here, we outlined that in the CCl_4_ model of experimental cirrhosis, LSECs were able to cooperate in the induction of effector Th17 subpopulations and to overcome the tolerogenic Treg differentiation profile in these animals, even at higher rates than hepatic DCs and HMs in this context, probably due to their immediate exposure to spontaneous bacterial translocation episodes. The development of Th17 cells has been described in a variety of liver diseases [38]. IL-6 present in the inflammatory context, commonly boosted by bacterial translocation in cirrhosis [39], contributed to Th17 differentiation, skipping the Treg induction achieved under homeostatic conditions. Induced bacterial peritonitis in this model was not able to increase pro-inflammatory effector CD4+ T cells. In fact, the T CD4+ cell differentiation was even lowered. These results suggested either (i) the high immunogenicity of the CCl_4_ model per se [40], (ii) the described immune dysfunction associated with acute-on-chronic liver failure in cirrhosis [41], which, in turn, would be supported by the reduced bacterial internalizing capacity observed in cirrhotic iBP animals, or even (iii) an insufficient culture exposure to reach full clonal expansion.

On the other hand, and according to the lower percentage of cells expressing the CD25 activation marker (Figure 5D), the BDL model displayed a low effector CD4+ T cell induction by hepatic APCs and a reduction in Th17 cell expansion. This was probably supported by the inhibitory role of LSECtin through the interaction with its ligand CD44, expressed on activated T cells [42]. In agreement, LSECtin was highly expressed in LSECs in the BDL model, while it was reduced in CCl_4_-treated animals (Figure 1), although the mechanism preventing Th17 differentiation in BDL remained to be determined in further studies. Additionally, although changes in gut microbiome as a consequence of BDL have been described [43], the question remains on whether spontaneous bacterial translocation may not play such a relevant pathogenic role in the BDL model as in CCl_4_ or if it may induce alternative inflammatory pathways in BDL. In fact, microbiota dysbiosis in BDL mice has been reported to be similar to control mice, while dysbiosis in CCl_4_-treated mice is characterized by the increase in *Firmicutes* and *Actinobacteria* [6]. However, when bacterial peritonitis was induced by *E. coli* in BDL, effector Th1 and Th17 cells were then primed by LSECs, confirming bacterial translocation as a prominent source of immune activation.

While both CCl_4_ and BDL are commonly used experimental models of advanced chronic liver disease, several differences are present among the toxic and the cholestatic models [44]. The specific immune response to each protocol may, therefore, vary to some extent. Contrasting Th17 polarization in CCl_4_ and BDL models may constitute one of the main differences between the two experimental models of liver damage. While LSECs induced an effector Th17 subpopulation in the CCl_4_ model that failed to be further induced by iBP, they displayed a lower induction of this population in BDL that, in turn, was able to be primed under iBP conditions. Another interesting difference would be defined by the opposite CD86 expression profile in hepatic DCs between CCl_4_ and BDL models (Figure 4A). This might help explain DC’s discrete ability to promote a complete Th response in this setting.

Finally, all immune features of hepatic APCs evaluated in this study were also explored in animals receiving norfloxacin to evaluate the immunomodulatory effect of this quinolone in both models. Its administration in both CCl_4_ and BDL models resulted in a smooth transcriptional modulation of innate receptor genes in all APCs compared to cirrhotic rats without norfloxacin and a global downregulation when compared with cirrhotic animals challenged with bacteria (Figure 7). Despite this attenuation, APCs from animals with norfloxacin preserved their functionality. LSECs modulated their ability to express co-stimulatory molecules in response to norfloxacin to maintain a highly active phagocytic capacity, even above other APCs, and to activate CD4+ T cell and to induce an increment of FoxP3 tolerogenic Th CD4+ cells, markedly immunomodulating Th17 response in CCl_4_-treated animals, whereas this effect was more attenuated in BDL model, as commented probably related to a slighter effect of bacterial dysbiosis and translocation in this latter model (Figure 8). The immunomodulatory properties of quinolones have been considered in the past, although specific mechanisms of actions remain elusive. Investigators have proposed different interactions with quinolones, such as their effect on intracellular intermediaries (cyclic adenosine monophosphate, protein kinase A, or phosphodiesterases) and on signal transduction and intracellular transcription factors like NF-kB, or their ability to inhibit topoisomerase II, leading to mammalian stress response [45]. In particular, the use of norfloxacin in cirrhosis has evidenced a soluble inflammatory control activity and immune regulation on neutrophils and Tregs [46,47].

Several study limitations need to be acknowledged. We could only provide indirect evidence of Th17 expansion, as specific experiments to understand the molecular functional mechanism were not carried out. Although the adaptive CD4^+^ T cell response regulation by innate receptors, such as LSECtin, has been proposed in acute liver injury [38], the mechanisms leading to the specific increase in the Th17 subpopulation in cirrhosis remain to be elucidated. We could hypothesize that these would include both intracellular signaling pathways and epigenetic regulation, with implications from the therapeutic perspective. Secondly, LSEC capillarization after liver damage might affect CD32b expression and, therefore, underestimate the number of LSECs collected. Finally, despite high purity populations of LSECs were achieved by adhesion, a residual contribution of contaminating cells could not be discarded. To minimize it, restricting timing in adhesion and washout procedures were followed [20].

In summary, the present study outlined several immune features of LSECs. In addition to their known role in driving activation of cytotoxic T cells or in the induction of tolerogenic CD8+ and CD4+ T cell responses, the modulated expression of innate receptors in LSECs and their participation in bacterial uptake during liver damage highlighted their features as liver APCs, beyond other physiological functions previously studied on this cell type in cirrhosis. LSECs ability to stimulate a pro-inflammatory CD4+ Th17 cell expansion in CCl_4_ and their differential response to an acute bacterial challenge in the context of cirrhosis pointed to specific inflammatory modulating mechanisms in experimental models of liver disease.

## Supplementary Materials

The following are available online at https://www.mdpi.com/2073-4409/9/5/1227/s1, Figure S1: Study design. Figure S2: APCs isolation and purity. Figure S3: Statistical differences in APCs’ mRNA expression. Figure S4: Flow cytometry of co-stimulatory molecules and isotype-matched controls. Figure S5: Gating strategy for Th subpopulation selection. Figure S6: Profibrogenic gene expression levels in CCl_4_ and BDL rats. Figure S7: Experimental work on female animals. Figure S8: Transcriptional profile of innate receptors, internalization assays, and Th differentiation markers in female rats. Table S1: Main role of receptors evaluated in hepatic APCs. Table S2: Primer-pair sequences used in the study.
