# Supplementary material for: Liver Sinusoidal Endothelial Cells Contribute to Hepatic Antigen-Presenting Cell Function and Th17 Expansion in Cirrhosis
Source: Cells. 2020 May 15;9(5):1227. doi: 10.3390/cells9051227 (PMC7290576; doi:10.3390/cells9051227)

Supplementary Figure 2. APCs isolation purity as determined by flow cytometry analysis specific labeling with αOX-62 for DCs, αCD68 for HMs and αCD32b for LSECs in control rats. A) Selection of single and live cell populations. Gating strategy for cell quantification before (B) and after (C) APCs purification protocol. Cell purity was confirmed by flow cytometry-specific labeling with αMHC-II for DCs, αCD163 for HMs, and αCD32b for LSECs.

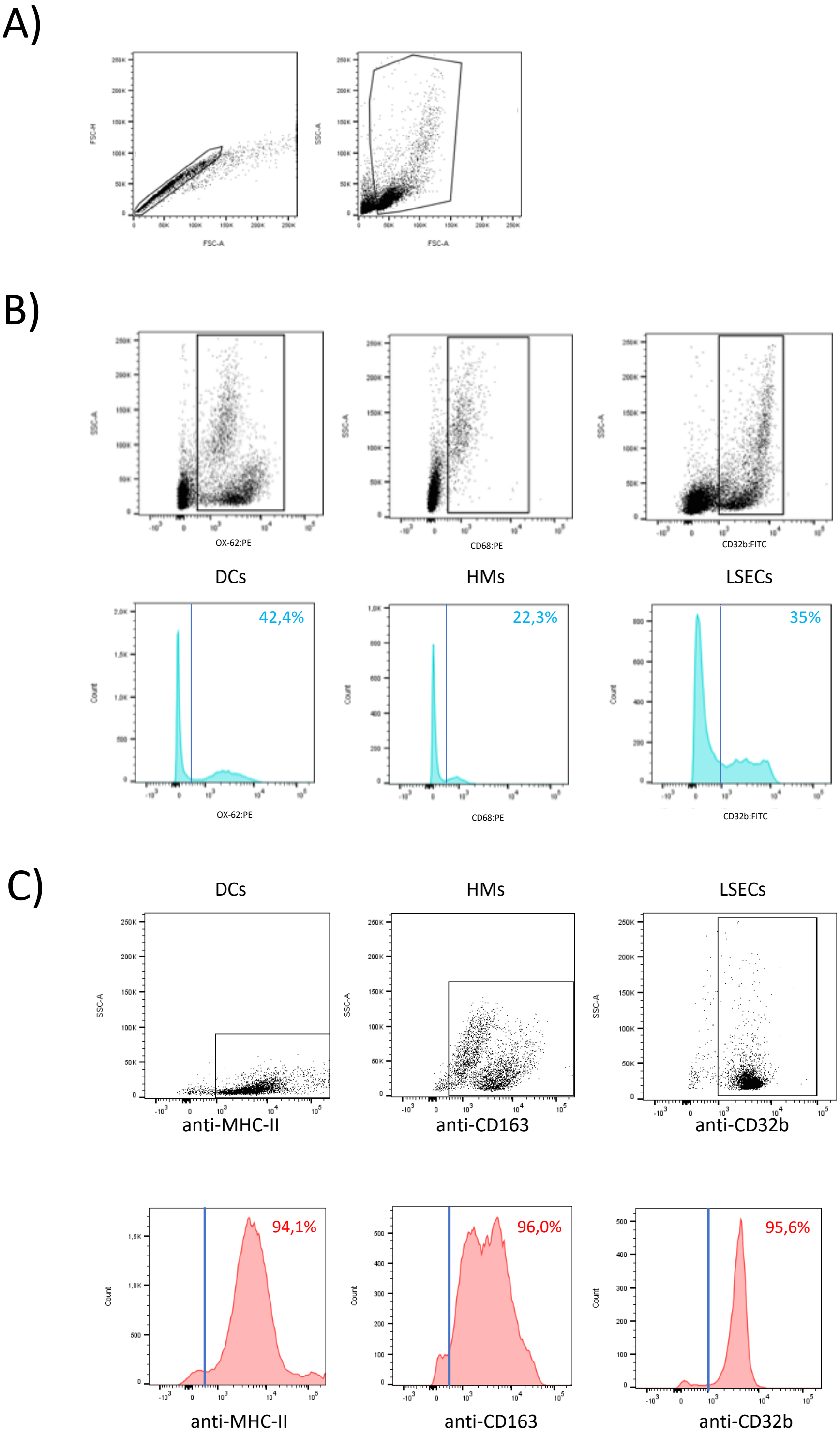

Supplement: Supplementary file 1 [file cells-09-01227-s001.zip › Figure S2.pdf]
