# Supplementary material for: Liver Sinusoidal Endothelial Cells Contribute to Hepatic Antigen-Presenting Cell Function and Th17 Expansion in Cirrhosis
Source: Cells. 2020 May 15;9(5):1227. doi: 10.3390/cells9051227 (PMC7290576; doi:10.3390/cells9051227)
Supplement: Supplementary file 1 [file cells-09-01227-s001.zip › Figure S3.pdf]

Supplementary Figure 3. *P* values for statistically significant differences between groups for each APC according to the Mann-Whitney U test are represented. Differences between APCs were analysed according to Bonferroni correction for multiple comparisons and represented with asterisk when statistically significant.

CCl4 vs Mineral oil control

	DCs	HMs	LSECs	
SCARB1				
MSR1	0.016			
CD206				
LSECtin				
TLR-2			0.015	*
TLR-4			0.008	*
CD16		0.009	0.012	
CD14		0.014		*
C5aR				
C3			0.022	*
CR1				
CR4				
CRIg				
CD40			0.018	
CD80		0.017		
CD86				
CD11a	0.009			
CD11b				
CD11c	0.012	0.014		*
FolR2		0.010		

iBP vs Naïve control

	DCs	HMs	LSECs	
SCARB1	0.017			
MSR1	0.015	0.032		
CD206				
LSECtin				
TLR-2			0.009	*
TLR-4			0.012	*
CD16				
CD14				
C5aR				
C3				
CR1	0.03			
CR4				
CRIg				
CD40				
CD80				
CD86				
CD11a				
CD11b				
CD11c	0.022			
FolR2		0.033		

CCl4+iBP vs non-cirrhotic iBP

	DCs	HMs	LSECs
SCARB1			
MSR1			0.025
CD206			
LSECtin			
TLR-2			0.03
TLR-4	0.011		0.02
CD16	0.021		0.016
CD14			
C5aR			
C3			
CR1			
CR4			
CRIg			
			0.028
CD40			
CD80			
CD86			
CD11a			
CD11b			
CD11c			
	0.035		
FolR2			

CCl4+iBP vs CCl4

DCs	HMs	LSECs		
				SCARB1
				MSR1
				CD206
		0.03		LSECtin
				TLR-2
		0.025		TLR-4
0.01			*	CD16
				CD14
				C5aR
		0.022		C3
				CR1
				CR4
				CRIg
				CD40
				CD80
				CD86
0.008				CD11a
0.012				CD11b
		0.022		CD11c
0.015			*	FolR2

BDL vs Sham-operated control

	DCs	HMs	LSECs	
SCARB1			0.035	
MSR1				
CD206		0.021	0.012	
LSECtin			0.018	
TLR-2		0.016	0.022	
TLR-4		0.009	0.008	
CD16			0.031	
CD14				
C5aR				
C3	0.02			
CR1	0.018	0.017		
CR4		0.012		*
CRIg			0.015	
CD40		0.008		
CD80				
CD86				
CD11a			0.014	
CD11b		0.010	0.012	
CD11c				
FolR2			0.013	

BDL+iBP vs non-cirrhotic iBP

	DCs	HMs	LSECs	
SCARB1		0.03		
MSR1			0.008	
CD206				
LSECtin	0.014	0.016		
TLR-2	0.035	0.028	0.018	
TLR-4	0.021	0.010	0.014	
CD16				
CD14	0.012	0.028	0.025	*
C5aR		0.011		
C3				
CR1	0.038	0.015	0.014	
CR4		0.008	0.010	
CRIg				
CD40		0.007		
CD80	0.016			
CD86				
CD11a			0.02	
CD11b		0.018	0.011	
CD11c	0.032			
FolR2	0.022		0.010	

BDL+iBP vs BDL

DCs	HMs	LSECs		
		0.008		SCARB1
		0.02		MSR1
				CD206
	0.013	0.018		LSECtin
				TLR-2
		0.012		TLR-4
0.09				CD16
0.010	0.026		*	CD14
0.008				C5aR
0.012				C3
0.010				CR1
0.010		0.021		CR4
				CRIg
	0.022			CD40
				CD80
				CD86
0.016	0.014	0.016	*	CD11a
				CD11b
	0.014			CD11c
0.018		0.017	*	FolR2

CCl4+Nflx vs CCl4

	DCs	HMs	LSECs
SCARB1			
MSR1			
CD206			
LSECtin			
TLR-2			
TLR-4			
CD16			0.025
CD14			
C5aR			
C3			
CR1			
CR4			
CRIg			
CD40			
CD80		0.035	
CD86			
CD11a			
CD11b			
CD11c			
FolR2			0.038

CCl4+Nflx vs CCl4+iBP

	DCs	HMs	LSECs
SCARB1			
MSR1			
CD206			
LSECtin			
TLR-2			
TLR-4			
CD16	0.022		
CD14			
C5aR			
C3		0.015	
CR1			
CR4			
CRIg			0.028
CD40	0.025		
CD80			
CD86			
CD11a			
CD11b	0.03		
CD11c			
FolR2			

BDL+Nflx vs BDL

	DCs	HMs	LSECs
SCARB1			
MSR1		0.030	
CD206		0.035	
LSECtin			0.02
TLR-2			
TLR-4		0.025	
CD16			
CD14			
C5aR			
C3			
CR1			
CR4			
CRIg			
CD40			
CD80			
CD86			
CD11a			
CD11b			
CD11c			
FolR2			

BDL+Nflx vs BDL+iBP

DCs	HMs	LSECs	
		0.030	SCARB1
		0.022	MSR1
			CD206
			LSECtin
			TLR-2
	0.014	0.018	TLR-4
0.022			CD16
			CD14
			C5aR
			C3
		0.035	CR1
		0.038	CR4
			CRIg
	0.033		CD40
			CD80
			CD86
0.032			CD11a
			CD11b
		0.026	CD11c
	0.025		FolR2
