# Supplementary material for: Liver Sinusoidal Endothelial Cells Contribute to Hepatic Antigen-Presenting Cell Function and Th17 Expansion in Cirrhosis
Source: Cells. 2020 May 15;9(5):1227. doi: 10.3390/cells9051227 (PMC7290576; doi:10.3390/cells9051227)

Supplementary Figure 4. Cell surface expression of costimulatory molecules and their respective matched isotype control.

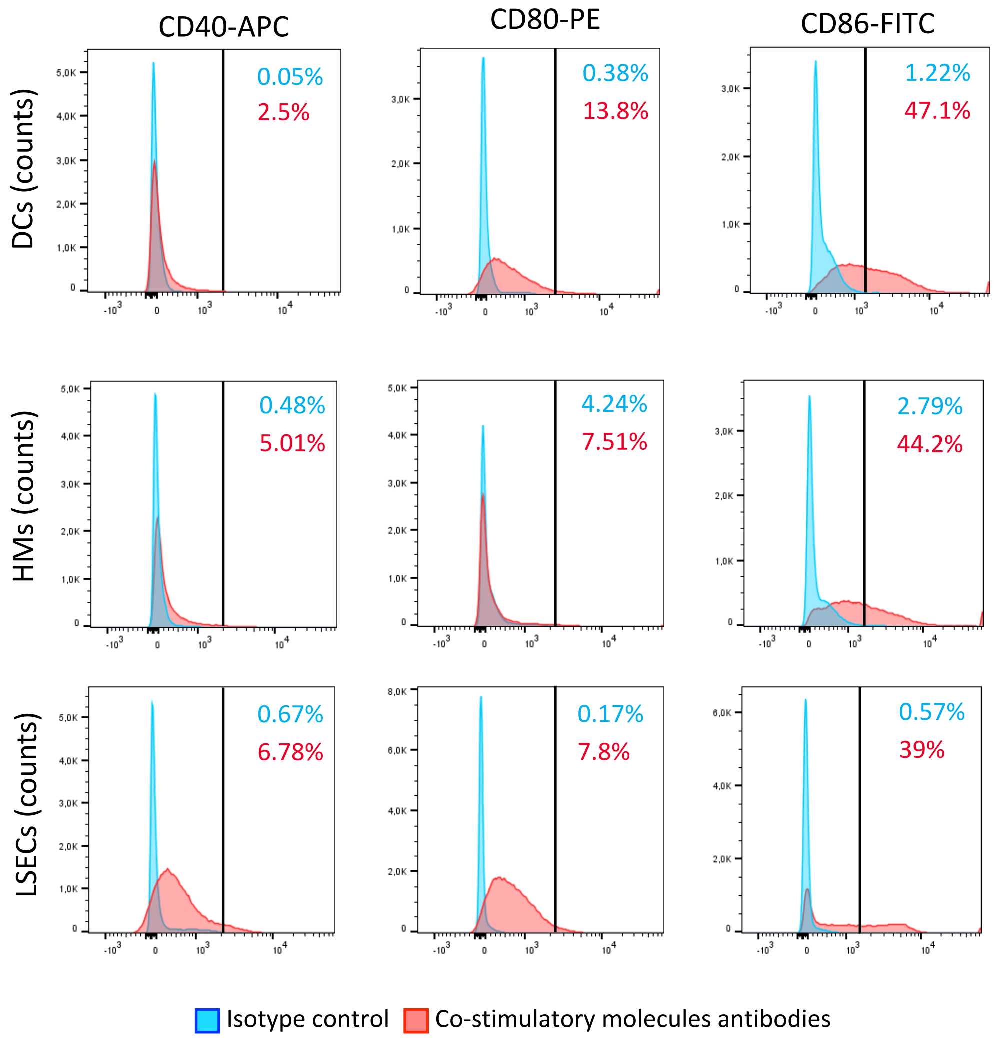

Supplement: Supplementary file 1 [file cells-09-01227-s001.zip › Figure S4.pdf]
