# Supplementary material for: Liver Sinusoidal Endothelial Cells Contribute to Hepatic Antigen-Presenting Cell Function and Th17 Expansion in Cirrhosis
Source: Cells. 2020 May 15;9(5):1227. doi: 10.3390/cells9051227 (PMC7290576; doi:10.3390/cells9051227)

Supplementary Figure 5. Gating strategy for Th subpopulation selection after surface/intracellular labeling with αCD4 and αIFN-g for Th1, αIL-4 for Th2, αIL-17 for Th17 and Foxp3 for Treg evaluation.

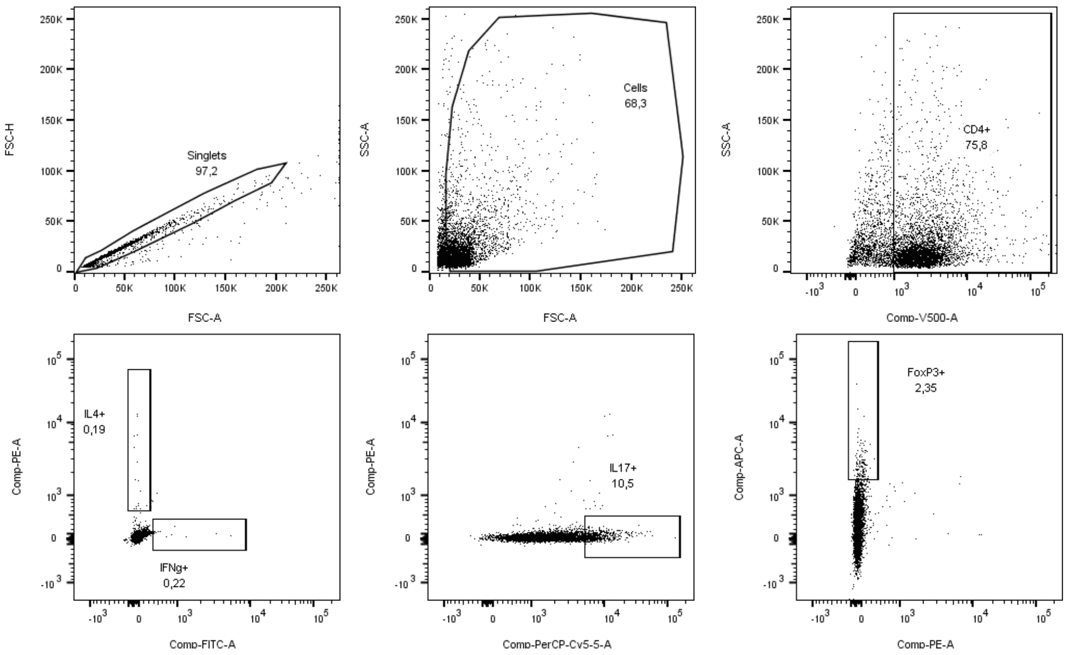

Supplement: Supplementary file 1 [file cells-09-01227-s001.zip › Figure S5.pdf]
