# Supplementary material for: Liver Sinusoidal Endothelial Cells Contribute to Hepatic Antigen-Presenting Cell Function and Th17 Expansion in Cirrhosis
Source: Cells. 2020 May 15;9(5):1227. doi: 10.3390/cells9051227 (PMC7290576; doi:10.3390/cells9051227)

Supplementary Figure 6. Profibrogenic gene expression levels in CCl_4 and BDL rats. Mean ± standard deviations are represented. *p<0.01 compared with controls. Col1a1: Collagen1 alpha 1; TIMP1: tissue inhibitor metalloproteinase 1; TGFb: transforming growth factor beta.

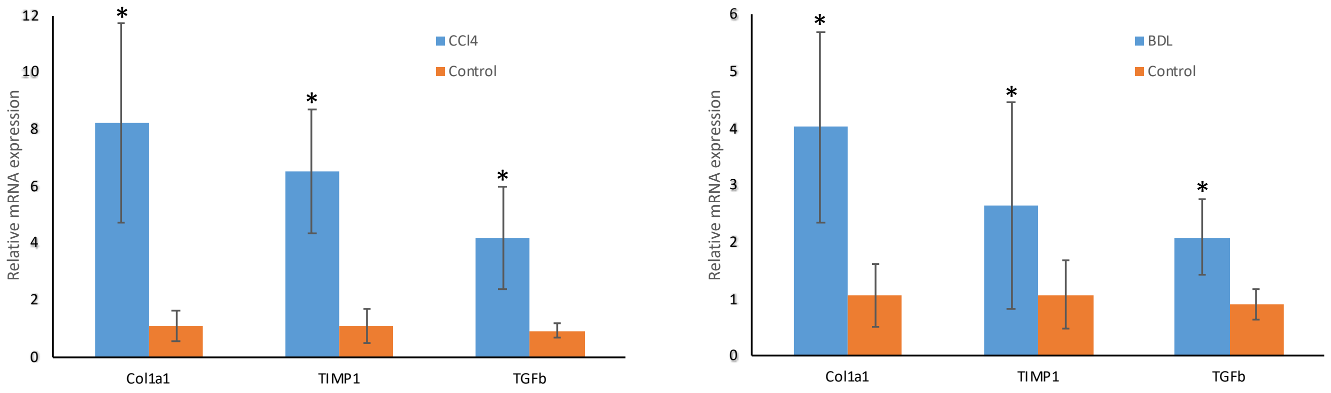

Supplement: Supplementary file 1 [file cells-09-01227-s001.zip › Figure S6.pdf]
