# Supplementary material for: Liver Sinusoidal Endothelial Cells Contribute to Hepatic Antigen-Presenting Cell Function and Th17 Expansion in Cirrhosis
Source: Cells. 2020 May 15;9(5):1227. doi: 10.3390/cells9051227 (PMC7290576; doi:10.3390/cells9051227)

Supplementary Figure 7. A) Transcriptional profile representation of innate receptors in antigen presenting cells from CCl_4 and BDL female animals in different experimental conditions. Differential expression of up (*red*) and down-regulated (*blue*) genes is scaled according to the color depicted. B) Internalization of *E. coli* by hepatic APCs from different study female animals groups. C) Differentiation markers intracellular expression in T CD4+ cells after co-culture with female hepatic APCs. Mean values ± standard deviation of percentage of T CD4+ cells expressing Foxp3 (Treg), IFNg (Th1), IL-4 (Th2) and IL-17 (Th17) are represented. * *p<0.01* compared to controls.

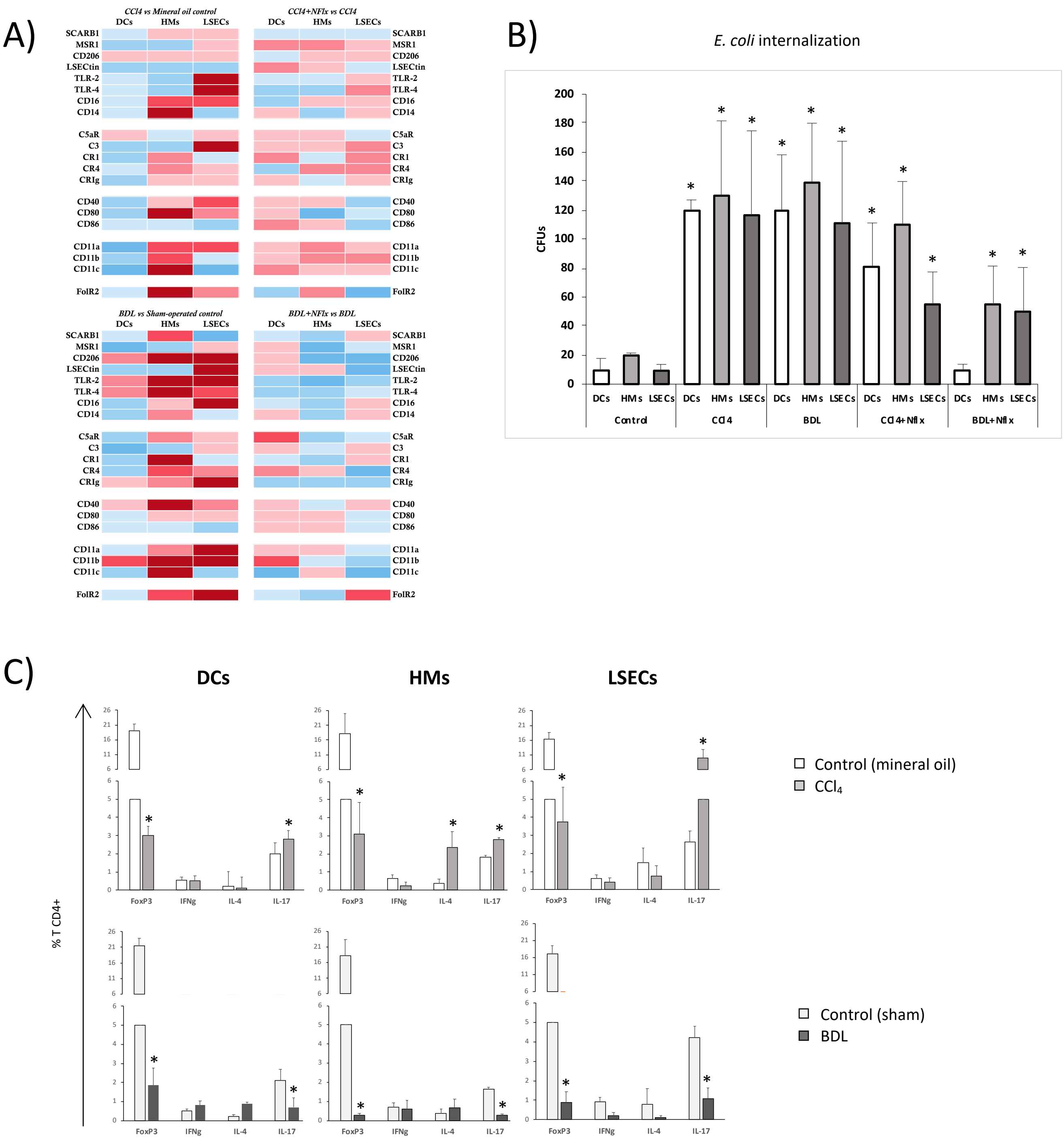

Supplement: Supplementary file 1 [file cells-09-01227-s001.zip › Figure S7.pdf]
