# Supplementary material for: Liver Sinusoidal Endothelial Cells Contribute to Hepatic Antigen-Presenting Cell Function and Th17 Expansion in Cirrhosis
Source: Cells. 2020 May 15;9(5):1227. doi: 10.3390/cells9051227 (PMC7290576; doi:10.3390/cells9051227)
Supplement: Supplementary file 1 [file cells-09-01227-s001.zip › Tables S1 and S2 R1.docx]

**Supplementary Table 1**. Main role of receptors evaluated in liver APCs. Col1a1: Collagen1 alpha 1; C: Complement; CR: Complement Receptor; CRIg: Complement receptor of the Immunoglobulin superfamily; CD: cluster differentiation; FOLR2: folate receptor 2; LSECtin: Liver and lymph node sinusoidal endothelial cell C-type lectin; MSR1: macrophage scavenger receptor 1; SCARB1: scavenger receptor class B member 1; TGFb: transforming growth factor beta; TIMP1: tissue inhibitor metalloproteinase 1; TLR: Toll-like receptor.

| **Marker** | **Function** |
| --- | --- |
| SCARB1 | Scavenger Receptor Class B Member 1/CD36  High Density Lipoprotein cholesterol (HDL) membrane receptor |
| MSR-1 | Macrophage Scavenger Receptor 1/ Scavenger receptor A  Binding of lipoteichoic acid, LPS, as well as acetylated/oxidized low-density lipoprotein (LDL) |
| CD206 | Mannose receptor (MR)  Scavenging of mannoglycoproteins and microorganisms with mannan-coated cell wall. |
| LSECtin | Liver and lymph node sinusoidal endothelial cell C-type lectin/CLEC4G  Pathogen receptor for Ebola virus and SARS coronavirus. Receptor for CD44 molecule. |
| TLR-2 | Toll-like receptor 2  Receptor for a variety of bacterial, viral and fungal antigens. |
| TLR-4 | Toll-like receptor 4  Receptor for lipopolysaccharide, HSP60, HSP90 or fibrinogen. |
| CD16 | Type III Fcγ receptor  Bind to the Fc portion of IgG antibodies |
| CD14 | Coreceptor for bacterial lipopolysaccharide |
| C5aR | Complement C5a Receptor |
| C3 | Complement component C3 |
| CR1 | Complement receptor 1/ CD35  Negative regulator of complement cascade |
| CR4 | Complement receptor 4/ CD11c/CD18 heterodimer  Facilitates phagocytosis of C3 fragments |
| CRIg | Mediates the phagocytosis of opsonized particles and pathogens. Receptor for the complement component 3 fragments C3b. |
| CD40 | Mediator of antigen presenting cells for interaction with T cells |
| CD80 | B7.1/Mediator of antigen presenting cells for interaction with T cells |
| CD86 | B7.2/Mediator of antigen presenting cells for interaction with T cells |
| CD11a | ITGAL/Heterodimerizes with CD18  Integrin adhesion molecule |
| CD11b | ITGAM/Heterodimerizes with CD18/Integrin adhesion molecule |
| CD11c | ITGAX/Heterodimerizes with CD18/Integrin adhesion molecule |
| FolR2 | Folate Receptor Beta/High affinity receptor for folic acid |

**Supplementary Table 2**. Primer pair sequences used in the study. Col1a1: Collagen1 alpha 1; C: Complement; CR: Complement Receptor; CRIg: Complement receptor of the Immunoglobulin superfamily; CD: cluster differentiation; FOLR2: folate receptor 2; LSECtin: Liver and lymph node sinusoidal endothelial cell C-type lectin; MSR1: macrophage scavenger receptor 1; SCARB1: scavenger receptor class B member 1; TGFb: transforming growth factor beta; TIMP1: tissue inhibitor metalloproteinase 1; TLR: Toll-like receptor.

| β2−microglobulin | CGTGCTTGCCATTCAGAAAAC |
| --- | --- |
|  | GAGGTGGGTGGAACTGAGAC |
| Col1a1 | TCCGGCTCCTGCTCCTCTTA |
|  | GTATGCAGCTGACTTCAGGGATGT |
| C3 | GACCTGCGACTGCCCTACTCT |
|  | CTGATGAAGTGGTTGAAGACG |
| C5aR | CAGGACATGGACCCCATAAGTA |
|  | CAGGAACACGGCCAAGTAGA |
| CD206 | TGGGTTTGCTGAAGAAGAGAA |
|  | CATGTGATAAGTGACAAATGCTTG |
| CD11a | TCTCCTTCCGAAAAGTGGAG |
|  | CCTCGCAGCTCACAGGTATT |
| CD11b | ACTCTGATGCCTCCCTTGG |
|  | CCTGGACACGTTGTTCTCAC |
| CD11c | GCCTCGAGACTGGAGATCAT |
|  | GGAGAGCTGGGAGCCAGT |
| CD14 | AAAGAAACTGAAGCCTTTCTCG |
|  | AGCAACAAGCCGAGCATAA |
| CD16 | CAGCTAGACGTCCATGCAGA |
|  | TGGCATCTCAGACGAATGG |
| CD40 | TGCCAACTCAATCAAGGGCT |
|  | TCCTTTGGTTTCTTGACCACCT |
| CD80 | ACACGACGTACGACCACTTC |
|  | GCAGCGTCGGAATTTGACAG |
| CD86 | AGACATGTGTAACCTGCACCAT |
|  | AAGCCCGTGTCCTTGATCTG |
| CR1 | GTGAGATACCCCCAAGCATTCC |
|  | CCACCAGGTTAAAGAGCTTCTTCC |
| CR4 | GGATCCAGCAGTTTCGGAAG |
|  | ACTGGACATGGGTCGTGGAA |
| CRIg | AACACCTCAGGGGACCACTA |
|  | GATCAAGATTATGGCAAAGATCG |
| FOLR2 | GACAAGCTGCATGACCAGTG |
|  | AGACGGGAGTTGTCCTTGTG |
| LSECtin | CAACAAGATTCAAGGCTACCG |
|  | GGCTCTCCAGAGTTCCAGTG |
| MSR1 | CTGGTGTTCCAGGTGCAAG |
|  | AAGCCAACTGGTCCCTGAT |
| SCARB1 | TCGAACAGAGCGGGATGATG |
|  | TTGGCTTCTTGCAGTACCGT |
| TGFb1 | AGAGGTCACCCGCGTGCTAA |
|  | TCCCGAATGTCTGACGTATTGA |
| TIMP1 | TCCTCTTGTTGCTATCACTGATAGCTT |
|  | CGCTGGTATAAGGTGGTCTCGTT |
| TLR2 | TCCTGAAGCTGTTGCGTTAC |
|  | TTGAGGGTGCAGTGATCAAA |
| TLR4 | CGCTTTCAGCTTTGCCTTCA |
|  | CTCCAGAAGATGTGCCTCCC |
